# Context-specific close-range “hoo” calls in wild gibbons (*Hylobates lar*)

**DOI:** 10.1186/s12862-015-0332-2

**Published:** 2015-04-08

**Authors:** Esther Clarke, Ulrich H Reichard, Klaus Zuberbühler

**Affiliations:** Evolutionary Anthropology Research Group, Dawson Building, Durham University, Durham, DH1 3LE UK; School of Psychology and Neuroscience, University of St Andrews, St Andrews, KY16 9JP UK; Department of Anthropology and left for Ecology, University of Southern Illinois, Carbondale, USA; Cognitive Science Centre, University of Neuchâtel, Neuchâtel, Switzerland

**Keywords:** Referential communication, Primate vocalisations, Hylobatidae, Ape communication, Language evolution

## Abstract

**Background:**

Close range calls are produced by many animals during intra-specific interactions, such as during home range defence, playing, begging for food, and directing others. In this study, we investigated the most common close range vocalisation of lar gibbons (*Hylobates lar*), the ‘hoo’ call. Gibbons and siamangs (family Hylobatidae) are known for their conspicuous and elaborate songs, while quieter, close range vocalisations have received almost no empirical attention, perhaps due to the difficult observation conditions in their natural forest habitats.

**Results:**

We found that ‘hoo’ calls were emitted by both sexes in a variety of contexts, including feeding, separation from group members, encountering predators, interacting with neighbours, or as part of duet songs by the mated pair. Acoustic analyses revealed that ‘hoo’ calls varied in a number of spectral parameters as a function of the different contexts. Males’ and females’ ‘hoo’ calls showed similar variation in these context-specific parameter differences, although there were also consistent sex differences in frequency across contexts.

**Conclusions:**

Our study provides evidence that lar gibbons are able to generate significant, context-dependent acoustic variation within their main social call, which potentially allows recipients to make inferences about the external events experienced by the caller. Communicating about different events by producing subtle acoustic variation within some call types appears to be a general feature of primate communication, which can increase the expressive power of vocal signals within the constraints of limited vocal tract flexibility that is typical for all non-human primates. In this sense, this study is of direct relevance for the on-going debate about the nature and origins of vocally-based referential communication and the evolution of human speech.

## Background

Primate vocal behaviour has been extensively studied, in recent years often with the aim of examining the cognitive underpinnings and evolutionary relationship to human language. Although language is a uniquely human behaviour, it is likely to have evolved from precursors in the primate lineage, some of which may still be detectable in the vocal behaviour of extant primates [1]. One important candidate for such a precursor is the ability to produce context-specific calls, a prerequisite to referential communication during which an actor refers a recipient’s attention to an external event. In animal communication, this is sometimes known as ‘functionally referential’ communication because it is usually not known whether or not such communication is intentional [2]. The classic example is the predator alarm call system of vervet monkeys (*Chlorocebus aethiops)* that produce acoustically distinct calls for different classes of predators, such as eagles, snakes and leopards [3,4]. Because vervet alarm calls can elicit distinct and adaptive anti-predator behaviours in listeners, even in the absence of a predator, it has been argued that the calls are meaningful to other monkeys, with some resemblance to human words or phrases [5]. More recently, functionally referential calling behaviour also has been described for other species of monkeys [6-9], apes [10-14], dogs [15], dolphins [16], and birds such as fowl [17], jays [18] and chickadees [19].

Overall, context-specific calling behaviour appears to be widespread in animal communication, presumably because the selection pressure to attend to and understand context-specific calls is very strong, especially in evolutionarily urgent situations. In addition, there is good evidence for call comprehension between different species of primates [20,21], between primates and birds [22] and between primates and other mammals [23], suggesting that such phenomena are driven by a generalised cognitive mechanism that is widely available to animals. Whether or not such abilities are relevant for understanding language evolution has triggered much debate with no real consensus [24,25]. Nevertheless, the comparative study of animal communication, especially across non-human primates, is one of the most useful tools to make progress and address open questions about human language evolution [26].

From an evolutionary perspective, context-specific communication is puzzling, mainly because it is not immediately clear why callers should provide accurate information about external events that they have witnessed. This is especially the case if the signals are costly to the caller, for example by attracting the attention of a predator or competitors, as in the case of alarm and food calls. Kin selection offers a plausible explanation [27], but this is relevant only if the caller can directly or indirectly benefit genetic relatives and enhance their chances of survival and reproduction. In primates this is often the case as most species tend to live in individualised social groups with a well-defined membership, suggesting that individuals regularly interact with individuals that are closely related to them [28].

Following the evolutionary logic of kin selection of context-specific calls, it is therefore surprising that primate vocal repertoires are generally small, consisting of only a handful of acoustically distinct call types [29], which is also true for human’s closest living relatives, the chimpanzees [30]. On the other hand, vocal repertoire sizes are often underestimated because many species appear to be able to generate subtle variations within some call types and because of combinatorial phenomena [31]. Both acoustic variants and signal combinations effectively increase a species’ repertoire size and therefore its expressive power. Recent examples are Diana monkey contact calls, which despite overall uniformity vary subtly according to social context [32], and chimpanzees and bonobos food-associated calls, which vary with food quality [13,33,34]. Subtle acoustic changes in otherwise identical call types may therefore function to counter the physical constraints of the generally inflexible non-human primate vocal tract.

In this study, we are interested in the variation of close-range ‘hoo’ calls of another ape species, the lar gibbon (*Hylobates lar*). Gibbons are mainly known for their loud and conspicuous songs, audible over long distances, which allow callers to communicate beyond their immediate home ranges in dense forest habitat [35,36]. In earlier work, we have presented observational evidence for functionally referential communication in these songs [37] and recent studies have compared the convergent development of patterns for human speech and human singing with gibbon song [38,39].

Lar gibbons also produce a number of soft call types, which so far have not attracted much attention. In a pioneering field study CR Carpenter [40] already alluded to close range calls in lar gibbons, which were described as “…low volume sounds” impossible to record with the technology available at the time. He described the entire vocal repertoire as consisting of nine distinct call types with five of them confined to within-group communication. These within-group calls functioned in cohesion, defence, play, begging, and directing others. JO Ellefson [41] labelled one call the “hoo” and described it as a “…broad pitch range soft to medium loud, being emitted singly or in short bursts of two or three per second” (p. 128). According to Ellefson (1974), hoos were produced in a variety of contexts, such as short distance separation from group members, responding to a human observer, encountering preferred food (“glug-hoo”), and during encounters with neighbouring groups (“conflict-hoo”). Although Ellefson speculated that hoos might show systematic acoustic variation across contexts, to our knowledge this hypothesis has never been tested empirically. In addition, JJ Raemaekers, PM Raemaekers and EH Haimoff [42] noted that hoos could also be part of *H. lar* songs, described as having a “…short, uninflected and steeply rising” shape, with a lower pitch and narrower frequency range compared to closely related “wa” calls. In a previous study [37] we confirmed these preliminary observations and suggested that “hoos” should be classed as a distinct “note” produced as a prelude to, as well as a part of, more complex songs. Because hoos are used in so many contexts there are reasons to believe that the acoustic distinctions alluded to by Ellefson [41] are context-specific and potentially meaningful to conspecifics. This study is a first step in determining whether these calls are consistently distinct and could therefore be used in functionally referential communication.

In this article, we focussed on lar gibbon hoos given in several main contexts: feeding, within group separation, alert (usually after hearing an inter-specific alarm call or some other disturbance), predator encounters, inter-group encounters, and as a prelude to duet singing. Given the adaptive value of context-specific vocal signals and the well documented comprehension abilities in primates, we predicted that we would find context-specific acoustic variations especially between some of the evolutionarily important events such as predator and other group encounters.

## Results

### Sample size

EC spent 117 days following the gibbons amounting to approximately 600 hours of observation. From these we extracted a total of 462 hoos from 14 males and eleven females across nine different contexts and subjected them to acoustic analyses. Sample sizes for alert, separation and snake contexts were too small to be included in the main analyses (Table 1). For the females, the inter-group encounter context was omitted since females often remained passive and spatially removed/peripheral during intergroup encounters, while males engaged and interacted with neighbouring individuals. More importantly, females typically did not produce hoo vocalisations during encounter situations.

**Table 1:** **Individual males and females in each context that contributed to the final analyses**

**Context**	**Number of individuals (n)**
Feeding	M = 5; F = 5
Inter-group encounter	M = 11; F = 0
Duet	M = 5; F = 7
Alert*	M = 1; F = 2
Separation*	M = 3; F = 3
Raptor	M = 7; F = 5
Tiger	M = 7; F = 7
Leopard	M = 8; F = 10
Snake*	M = 4; F = 4

M = Male, F = Female, * = Contexts that were excluded from the main analyses due to low sample sizes.

**Table 2:** **Statistical comparison of male and female hoo call contexts**

**Acoustic parameter (n value)**	**Sex**			**Context**		
	**df**	**F**	**p**	**df**	**F**	**p**
Duration (n = 379)	1.0, 25.0	0.9	0.334	5.0, 353.8	6.0	<0.001***
Peak frequency (n = 378)	1.0, 20.7	19.8	<0.001***	5.0, 354.5	11.8	<0.001***
Low frequency (n = 377)	1.0, 24.6	30.1	<0.001***	5.0, 361.9	16.3	<0.001***
Delta frequency (n = 377)	1.0, 23.2	0.7	0.422	5.0, 350.4	7.5	<0.001***
Inter-call interval (n = 300)	1.0, 19.3	0.17	0.7	5.0, 247.8	11.4	<0.001***
Intensity (n = 173)	1.0, 21.1	0.7	0.423	5.0, 156.6	7.8	<0.001***

All tests were LMMs with sex and context as fixed factors, and subject ID as a random factor; ***= significant difference; df: numerator, denominator.

We found that some acoustic parameters were easier to extract than others, which resulted in unequal sample sizes. For example, intensity was particularly difficult to measure, which yielded a smaller sample size than call duration, inter-call interval, and peak, low and delta frequency, which limited the number of possible comparisons.

### Sex differences

**Figure 1:**
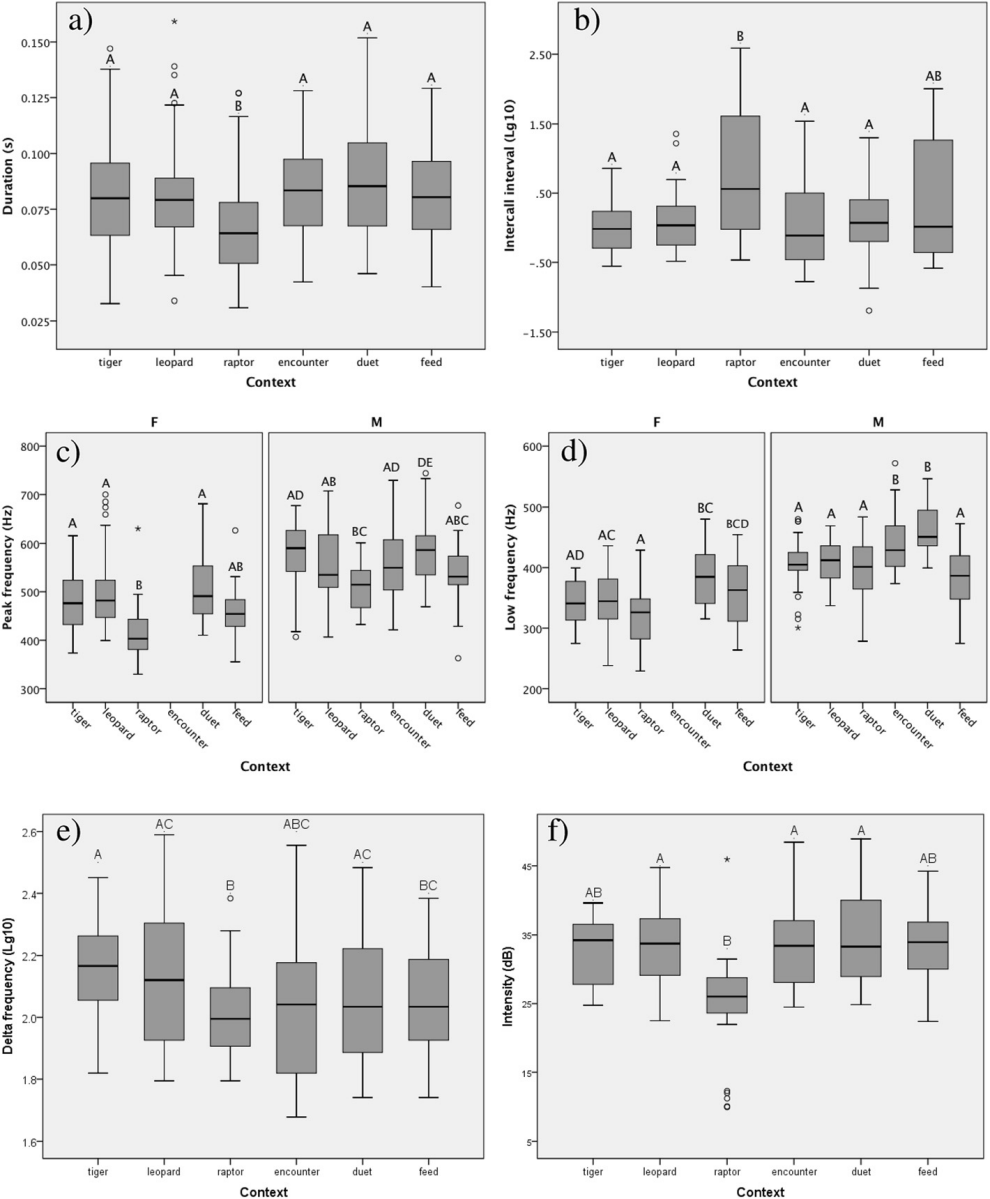
**a-f. Box plots of the mean values for each measured acoustic parameter (not accounting for individual identity).** Panel **a)** duration, **b)** intercall interval, **c)** peak frequency, **d)** low frequency, **e)** delta frequency, **f)** intensity. Error bars represent 95% CI. Different letters above box plots indicate significant differences between contexts. Where sex differences were found plots are split into two panels: F = female, M = male.

Gibbons lack an obvious sexual dimorphism in body size, possibly due to their sometimes monogamous grouping structure [43]. While Khao Yai gibbons show stable polyandrous groupings as well as monogamous ones [44], we do not predict any differences in vocal behaviour. We found that the average peak frequency of hoos was 521.9Hz ± 3.79SE, (n = 462) with an average duration of 0.08 s ±0.002SE (n = 462). Surprisingly, however, we also found that male hoos had significantly higher peak frequencies (F0) and low frequencies than female hoos (Table 2, Figure 1). No sex differences were found in other acoustic parameters.

**Table 3:** **Results of discriminant function analysis on hoo call contexts for males and females**

**Function**	**Eigenvalue (F/M)**	**% of Variance (F/M)**	**Wilks’ Lambda sig. tests of functions (F/M)**	**Correlation between variable and function (F/M)**
1	0.45/0.58	79.2/66.7	<0.001/<0.001 (Function 1–3)	intercall interval (−0.68/0.63)
2	0.12/0.23	20.8/26.7	<0.001/<0.001 (Function 2–3)	low frequency (0.86)/delta frequency (−0.67)
3	NA/0.06	NA/6.6	NA/0.017 (Function 3)	NA/low frequency (0.50)

F = female, M = male.

### Context-specific call variants

For both males and females, Linear Mixed Models (LMMs) revealed there were significant differences in acoustic parameters across the seven different contexts (Table 3, Figure 1).

**Figure 2:**
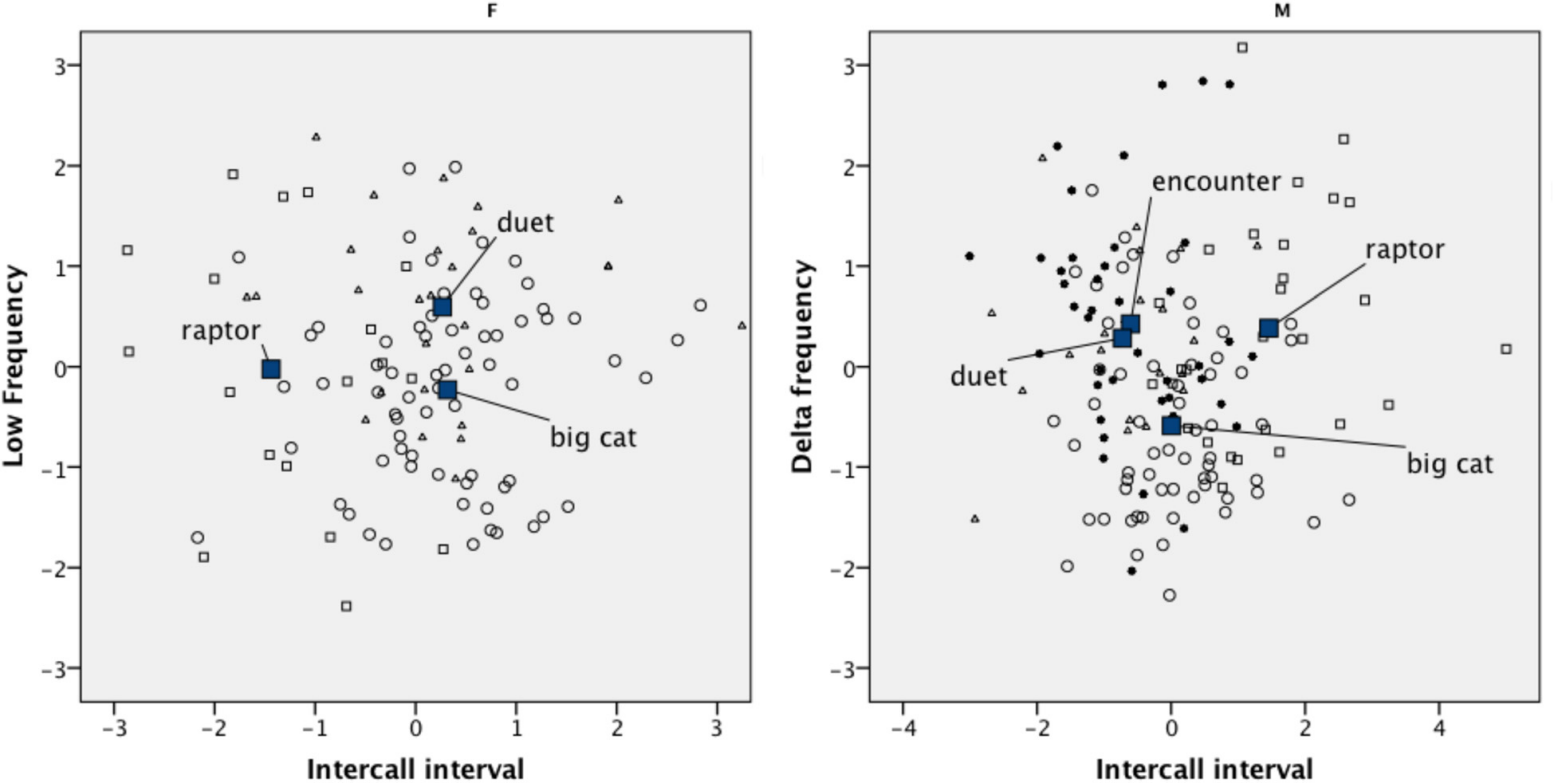
**Two scatter plots of functions 1 (intercall interval) and 2 (low frequency/delta frequency) as they explain the variation between the different hoo contexts in males (M) and females (F).** ○ = big cat; □ = raptor; ● = group encounter ∆ = duet; ■ = group centroid.

We then conducted a step-wise discriminant function analysis, entering the five most readily measurable acoustic parameters (peak frequency, low frequency, delta frequency, duration and inter-call interval) to determine whether the different contexts could be separated by the interactions between the measured variables. Since sex differences were frequency-related we conducted separate DFAs for males and females. Also, since hoos to leopard and tiger models did not differ from each other, these were pooled as “big cat” responses. Sample sizes for feeding hoos proved to be too small for the DFA when we split males and females. Similarly, intensity was excluded because sample sizes became too low (<20) when this variable was entered and sexes were split. Interestingly, intensity strongly predicted hoo context in initial analyses, making it particularly relevant for future study. The DFA results are summarised in Tables 3 and 4 and Figure 2.

**Table 4:** **Discriminant function analysis classification table for males and females**

	**Hoo context**	**Predicted Group Membership (F/M)**				**Total (F/M)**
		**big cat**	**raptor**	**encounter**	**duet**	
Count	big cat	62/47	3/3	NA/8	3/2	68/60
	raptor	9/11	10/15	NA/2	1/0	20/28
	encounter	NA/15	NA/2	NA/23	NA/4	NA/44
	duet	19/8	2/1	NA/8	6/3	27/20
%	big cat	91.2/78.3	4.4/5.0	NA/13.3	4.4/3.3	100.0
	raptor	45.0/39.3	50.0/53.6	NA/7.1	5.0/0.0	100.0
	encounter	NA/34.1	NA/4.5	NA/52.3	NA/9.1	100.0
	duet	70.4/40.0	7.4/5.0	NA/40.0	22.2/15.0	100.0

F = female, M = male; 67.8% (female) and 57.9% (male) of original grouped cases correctly classified.

**Table 5:** **Behavioural contexts recorded during production of hoo calls**

**Context**	**Definition**
Feeding	Individual is engaged in handling or consumption of an edible item
Separation	Individual is separated from the rest of the group, often during locomotion
Alert	Individual stares at a fixed point in space for five seconds or more, often accompanied by alarm calls by other species
Ground predator	Individual stares at real or experimentally presented predator on the ground. Predator models were a clouded leopard (*Neofelis nebulosa*), a tiger (*Panthera tigris*) and a snake (*Python reticulatus*)
Raptor	Individual has perceived a real or experimentally presented raptor. Real raptors were eagle owls, (*Bubo bubo)*. Raptor models were crested serpent eagles (*Spilornis cheela*)
Inter-group encounter	Individual is in visual contact with a neighbouring group
Duet	Individual sings with their mate

## Discussion

We investigated the close-range hoo calls of free-ranging lar gibbons in a natural forest habitat of North-eastern Thailand to establish whether there were acoustic differences between the various contexts they were emitted in. Close-range calls of wild gibbons have not received much attention nor have they previously been analysed in detail, in contrast to countless studies on their singing behaviour [42,45-47]. Results revealed that both adult males and females produced context-specific hoo calls, according to the following patterns. First, both sexes were not distinguishable in the acoustic structure of their hoo calls given in specific contexts. For example, measures of peak and low frequency showed that the hoos given in response to raptors were significantly lower than the hoos given as part of a daily duet song, for both males and females. Furthermore, raptor hoos showed significant differences in a number of acoustic parameters compared to many of the other contexts: they were significantly shorter in duration than duet and leopard hoos for both males and females and raptor hoo intensity was significantly lower than the duet hoos, leopard hoos and tiger hoos. In addition, inter-call intervals were significantly longer for raptor hoos than hoos in other contexts, such as tiger, duet, leopard (males and females), and encounter hoos (males only). In fact, raptor hoos were acoustically distinct from all other context, including leopards and tigers, suggesting that they can be classified as a predator-specific call variant. This is in contrast to gibbon loud vocal responses to other predators [37,48]. Due to the gibbons’ relatively small body size raptors are a real threat to infants and juveniles, but probably not to mature gibbons, which may explain the different anti-predator responses by adult gibbons to raptors and cats [48]. Raptor hoos are occasionally also emitted by non-adults and it would therefore be interesting to acoustically compare non-adult and adult hoos in a future study. Raptor hoos were less intense, more widely spread out, and of shorter duration, lower frequency and smaller frequency span than the other hoos, making them the least audible of all hoo variants. This is consistent with the interpretation that raptor hoos are given in circumstances the gibbons may perceive less threatening or to prevent attracting the attention of the predator. For example some passerine bird raptor alarm “seet” calls are difficult to localise [49]. Raptors hear best in the range of 1-4 kHz [50,51], with sensitivity to sounds below and above this being much poorer than primates. Of note, gibbon hoos are consistently below the 1 kHz threshold, with raptor hoos being the lowest frequency of all. The gibbons’ relatively cryptic responses to different predators is consistent with what has been reported in other primates [52,53].

We also found further differences between other hoo contexts. For example, duet hoos tended to be higher in frequency than the other contexts, significantly so when compared with feeding, leopard, tiger and raptor hoos (males) and raptor and tiger hoos (females). The delta frequency of the tiger and leopard hoos tended to be greater than in feeding hoos (females). For encounters, we only obtained recordings from the males, since females did not usually engage in inter-group encounters, but here we also found significant differences in frequency measures if compared to the tiger, leopard and feeding context and significant differences in delta frequency if compared to tiger hoos. The only two contexts that did not differ significantly from one another were tiger and leopard hoos, suggesting that callers perceived these two predators as belonging to the same class.

Apart from contextual effects we also found some significant sex differences in the acoustic structure of hoo calls, despite the absence of sexual dimorphism in this species. Overall, female hoo calls had a significantly lower peak frequency than male hoo calls, in line with an earlier study [42]. Male hoo low frequencies were also significantly higher than female hoos, but we found no differences in delta frequency or intensity between the sexes.

Overall, the data presented here show that the differences between males and females are mainly in terms of frequency-related parameters, while the way that both sexes vocalise to contexts is identical. Among mammals in general, males tend to have lower frequency voices than females, possibly due to larger body size [54] so our findings are surprising. In gibbons, female voices are lower in frequency than males, despite the absence of obvious differences in body size across gibbon species [43]. One possibility is that subtle differences in *H. lar* body mass may be responsible: males are 0.56 kg (9%) heavier than females [55], though we would expect the opposite relationship with voice frequency if this slight difference in mass was important. Another possibility is that testosterone levels are partly responsible; the singing voices of adult male gibbons with higher testosterone have higher F0 [56], and since males typically have higher testosterone than females, this may better explain our findings. However, the ontogeny of gibbon voice qualities and their relationship with steroid hormones in both sexes require further research.

A DFA revealed intercall interval separated raptor hoos from big cat and duet hoos (females and males), and also encounter hoos (males). Low frequency separated big cat and raptor from duet hoos (females) and delta frequency separated duet, encounter and raptor from big cats (males). Overall, intercall intervals separated big cat and raptor hoos (males and females), while frequency-related measures separated duet from big cat hoos (males and females). For all contexts, there was also considerable overlap, suggesting that other acoustic variables may also contribute to distinguishing call context. For example, intensity appeared to be important but for reasons explained earlier, we omitted it from the final DFA. Also, without being able to fully control for the effects of distance from vocaliser to microphone and amount of vegetation, which could attenuate vocal signals, we remain cautious in interpreting the role of intensity in gibbon hoo communication. A larger sample size of excellent quality hoo recordings would be necessary to explore this preliminary finding.

In future work, it will be important to carry out systematic playback experiments of different hoo variants to determine whether this call type functions as a referential system in the way that their long-distance songs do [37].

The hoo calls described in this paper are virtually indistinguishable to human observers and call variants became only apparent in statistical analyses. Gibbon hoos should therefore be classed as an example of an acoustically graded call type, alongside other primate vocalisations, such as chimpanzee pant hoots [14] or chacma baboon grunts [57]. Social variables, such as an individual’s physical condition or social status, might additionally influence some of the acoustic parameters, which could explain some of the overlap seen in Figure 2. Since hoos are produced in multiple contexts, the motivational states of the callers will likely vary between them, which is particularly the case in predator hoos and inter-group encounter hoos. Some authors interpret such findings as evidence that animal signals do not carry any “meaning” [58,59], but that they serve to *influence* rather than *inform* listeners, which subsequently can become conventionalised using linguistic pragmatics [58]. However, while attention-getting vocalisations may not be *designed* to inform, it is difficult to argue that they do not do so [60], which in our opinion make such findings relevant to understand the origins of “meaningful” communication, including language [26].

Given the obvious selective advantage of ‘labelling’ contexts with distinct signals, why do gibbons and other non-human primates not produce acoustically more discrete signals for different contexts, instead of the subtle acoustic variations within just one call type? We can think of the following possibilities. Firstly, non-human primates may generally be prevented from the required vocal tract control to produce more discrete signals, due to anatomical and neurological constraints, the classic argument to explain limitations in vocal flexibility and control in non-human primates [61]. Gibbons are not alone in this, as there is increasing evidence that subtle changes within the main basic call types can generate meaningful information (see [62]). Secondly, the acoustic variation seen in gibbon hoos (and in other primate vocalisation types) may simply be the basis of acoustic flexibility, similar to human speech, in which subtle acoustic parameters, like pitch, can be important carriers of meaning (e.g. Chinese or Thai: [63]).

Context-specific calling behaviour appears to be widespread and therefore was likely present in the ancestor of modern primates and humans. Similar abilities are found in birds whose distinctive vocal organs are very different, suggesting that context-specific graded vocal behaviour has evolved independently, as did vocal flexibility and vocal learning. Amongst the primates, gibbons are an especially interesting taxon because, like birds, they produce songs, and may have greater vocal tract control than other non-human primates [64]. More research on gibbon song is needed to describe its complexity and the cognitive factors associated with song production [23,37-39,48,64].

Comparing the vocalisations of non-human primates with human language is of interest because of shared phylogeny. So far, the main difference between human and non-human primates is largely in terms of flexibility in production, but less so in terms of comprehension [65-67]. However, ours and other authors’ research show that non-human primates have the capacity to generate considerable acoustic variability, within the constraints of their basic vocal repertoire, which widens their communicative power considerably.

## Conclusion

Lar gibbons reliably produce context-dependent hoo calls in different contexts, including foraging, predator detection, encountering neighbours, and in duet songs. Differences are not just between predator and non-predator contexts, but also within them. We carried out wide-spread acoustic comparisons and found a complex set of subtle spectral parameters that consistently discriminated contexts both within and between sexes. An acoustically unique class were the raptor hoos, which were clearly distinct from all other contexts, presumably designed to cope with avian predators. Our results are in line with other research that has emphasised the importance of graded call systems in communicating external events, such as chimpanzee pant hoots [14], several calls in the Barbary macaque repertoire [68] and chacma baboon grunts [57]. Playback experiments will be needed to determine whether different hoo calls are discriminated by recipients and whether they elicit appropriate responses, as it has been shown for other graded systems (baboons [69,70]; macaques [69,71]; vervet monkeys [72]). We propose that subtle contextual variation in otherwise homologous calls might be a way to increase the expressive power of non-human primate vocalisations where extensive vocal flexibility is not possible.

## Methods

### Study site and subjects

The study took place at Mo Singto in Khao Yai National Park, Thailand, approximately 130 km NE of Bangkok (101°229E, 14°269 N), and at an elevation of 730-860 m. The study groups consisted of between two and six individuals, typically an adult pair and their offspring, sometimes with more than one adult male. Males and females typically disperse from their natal group at around 10 years of age, about two years after reaching sexual maturity [73]. Lar gibbons are sexually monomorphic and of light or dark pelage colour, which is unrelated to sex or age. Data were collected from thirteen groups with all group members individually known (with the exception of one male from a neighbouring unhabituated group) and long-term social records were available for most individuals [74].

### Data collection and equipment

**Figure 3:**
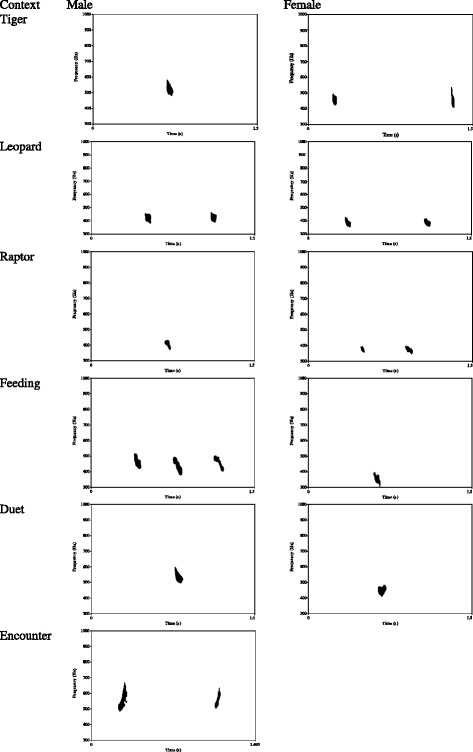
**Spectrographic illustration of hoos given in several contexts by males and females.**

Data were collected between April 2004 and July 2005 and between May and November 2007. Groups were located by listening to their morning duets or by encountering them at their sleeping sites chosen the previous evening. Groups were usually followed from the first encounter in the morning until they had located their evening sleeping tree, the time of which varied greatly depending on the season. During these focal follows, all hoo calls were recorded and their corresponding contexts noted (Figure 3, Table 5). To elicit the rare predator hoos, we presented a series of fake predators, in realistic poses, close to the ground (terrestrial predators: tiger, clouded leopard), 1-2 m above ground on a low branch (arboreal predator: reticulated python) as well as in the canopy (aerial predator: crested serpent eagle). For full methodological details of these experimental presentations, see Clarke et al. [37]. Only hoos emitted by adults (including young adult offspring still residing in their natal group) were included in the analysis to avoid possible age-related acoustic variation seen in other primates [75]. Recordings were made with a Sennheiser directional microphone with windshield (ME66) and a Sony DAT recorder (TCD-D7).

### Data analysis

For each context, we extracted the first five hoos of each call bout for measurements. This was based on the assumption that if calls functioned to transmit something about the event encountered by the caller, then this should already be conveyed acoustically in the early parts of an utterance. In some instances, not every hoo of the first five could be measured completely, usually due to poor recording quality or overlap with other sounds. In these cases, the additional calls beyond the first five were included in the analyses. For each context we collected recordings from at least five individuals of each sex. Exceptions include alert, separation and snake hoos (see Table 2).

All calls were transferred to a computer using Cool Edit 2000 and Adobe Audition 3.0 before analyses with PRAAT 5.0.29 using the following settings: *Spectrogram settings*: window length = 0.05 s; analysis method = Fourier; window shape = Gaussian; number of time steps = 1,000; number of frequency steps = 250; maximum dB/Hz = 100.0; pre-emphasis dB/oct = 6.0; dynamic compression = 0.0; *Intensity settings*: Averaging method = mean energy; subtract mean pressure checked; *Pulse settings*: Maximum period factor = 1.3; maximum amplitude factor = 1.6. The viewing window was approximately 0.3 s.

**Figure 4:**
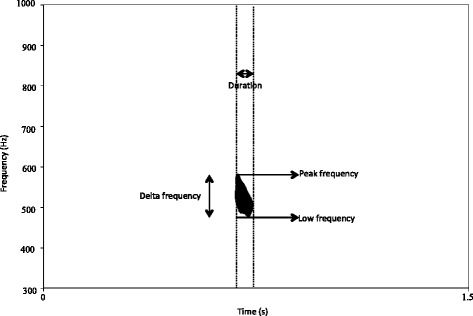
**Spectrographic illustration of a single hoo showing how acoustic measurements were taken.** Intercall interval was measured as the end of one hoo to the beginning of the next. Intensity was measured using PRAAT’s automated analysis scripts.

The following six acoustic parameters were extracted from calls based on ECs observations and MJ Owren and CD Linker [76]: duration (s); intensity (dB); peak frequency (Hz), low frequency (Hz), delta frequency (Hz) and inter-call interval (s) (see Figure 4 for illustrations; data available in LabArchives repository [77]).

Data were analysed and transformed where appropriate to meet the assumptions of parametric tests (delta frequency and intercall interval) using SPSS version20. Linear Mixed Models were used with subject identity as a random factor and included both sex and context as fixed factors. To explore the effects of context on acoustic parameters further, we used additional LMMs with identity as a random factor in pairwise analyses comparing different contexts using Bonferoni corrections for multiple comparisons.

A Discriminant Function Analysis (DFA) was also conducted to determine how the interaction between acoustic variables might predict membership to the different context groups.

### Availability of supporting data

The data set supporting the results of this article are available in the LabArchives repository: 10.6070/H49C6VCK
